# The relationship between hearing status, listening effort, and the need for recovery in employees of a manufacturing company

**DOI:** 10.1007/s00405-023-07898-x

**Published:** 2023-03-01

**Authors:** Hanneke E. M. van der Hoek-Snieders, Jan A. P. M. de Laat, Wouter A. Dreschler

**Affiliations:** 1 Department of Clinical & Experimental Audiology, Amsterdam University Medical Centre, Meibergdreef 9, 1105 AZ Amsterdam, The Netherlands; 2 Department of Audiology, Leiden University Medical Centre, Leiden, The Netherlands

**Keywords:** Hearing, Work, Screening, Fatigue

## Abstract

**Objective:**

Hearing screening can be used to detect hearing loss, but its value for identifying employees with work functioning difficulties is unclear. The objective of this study was to assess the association between the hearing status measured with an occupational hearing-in-noise screening test, Listening Effort (LE), and Need For Recovery (NFR) in employees of a manufacturing company, and to examine whether these associations depend on the perceived noise level at the workplace.

**Methods:**

Employees of coatings and paints manufacturing company were included. Their hearing status was assessed with an occupational hearing-in-noise screening test. An online survey was used to assess their LE, NFR, and the perceived noise level at the workplace. Responses from 143 employees were analyzed (mean age = 53 years) using hierarchical multiple regression analysis with the outcomes LE and NFR.

**Results:**

Regression analysis—with adjustments for gender, age, educational level, health status, pace/amount of work, job variety, and work pleasure—revealed that hearing status was significantly associated with LE, but the interaction between hearing status and the perceived noise level was not. Hearing status nor the interaction between hearing status and the perceived noise level was significantly associated with NFR.

**Conclusion:**

The results confirm that poorer hearing is associated with higher LE, but not with higher NFR. These associations were unrelated to the perceived noise level at the workplace. Therefore, the value of occupational hearing screening appears to be early identification of hearing loss in employees, but not identification of work functioning difficulties.

## Introduction

Hearing loss is a common condition in the working population, with higher prevalence with higher age and in populations that are exposed to occupational noise [1–4]. In the last decades, there has been an increasing interest in the impact of hearing loss on work functioning [5–9]. Even with mild hearing loss, the performance of auditory job tasks may take significantly more listening effort (LE), especially in noisy work environments [7, 10]. Sustained, effortful listening can be fatiguing and is associated with higher need for recovery (NFR) after work [9].

In line with earlier studies that indicate that high NFR is a predictor of negative work implications [11, 12], hearing loss has been found to hinder work participation. Some job tasks cannot be performed safely and effectively without sufficient hearing, which is for example the case in pilots, fire fighters, and locomotive engineers [13]. In these, but also in other occupations, work participation can also be under pressure when hearing loss is present. Hearing loss has been shown to be associated with reduced work productivity, higher levels of sickness leave due to mental distress, unemployment, and earlier retirement [5–8, 14].

In some workplaces, occupational hearing screening is applied to detect hearing loss in an early stage [15]. This serves several purposes. Occupational hearing screening can be used to ensure that employees can perform essential hearing-critical job tasks safely and effectively [13]. Also, noise -nduced hearing loss is preventable, and occupational hearing screening can contribute to take preventive measures [15]. In the Netherlands, occupational hearing screening is routinely offered to employees that work in workplaces with noise levels above 85 dBA. Finally, occupational hearing screening can stimulate employees to seek audiological help [16, 17], before they experience listening difficulties or other difficulties at work.

Several hearing tests have been proposed for screening purposes. Otoacoustic emissions (OAEs) objectively measure the outer hair cell function, pure tone audiometry evaluates the detection of sounds in a quiet environment, and speech-in-noise tests measure the ability to understand speech in a noisy environment [18]. Since difficulties with understanding speech in noise are the major complaint of people with hearing loss, speech-in-noise tests are considered most suitable for assessing functional hearing [18, 19]. The Occupational Ear Check (OEC) is a Dutch speech-in-noise test that has been developed for occupational screening purposes [20]. For both ears separately, the signal-to-noise ratio is assessed at which 50% of the speech stimuli can be identified correctly.

Many researchers have investigated the reliability and validity of speech-in-noise screening tests for identifying hearing loss, which is also the case for the OEC [18, 21]. However, it is yet unclear if the poorer screening outcomes are also associated with higher LE and higher NFR. Therefore, the value of occupational hearing screening for identifying employees with subjective listening difficulties and/or difficulties with work participation is still unclear.

In an earlier study, the outcome of a speech-in-noise test was significantly associated with LE, but not with the NFR [9]. This study included a clinical population and used a speech-in-noise test that is not designed for screening, but for clinical purposes. Also, the association between the outcome of the hearing screening, LE, and NFR might be different for employees that experience different noise levels at their workplace, but an interaction effect with occupational noise was not investigated thus far. Therefore, the aim of this study was to assess the association between the hearing status measured with an occupational hearing-in-noise screening test, LE, and NFR in employees, as well to examine whether these associations depend on the perceived noise level at the workplace.

## Methods

### Study design

This cross-sectional and observational study was conducted at a coatings and paints manufacturing company. We analyzed the outcome of an online survey and the outcome of an internet-based hearing screening. This screening is routinely administered at the company as part of a voluntary administered health check. The ethics Committee of the Academic Medical Center declared that no formal approval of the detailed protocol was required according to the Dutch Medical Research Involving Medical Subjects Act (No. W18_369 # 18.421).

### Population and procedures

With consent of the company’s management, information about this study was provided in the period from December 2020 to February 2022 at the company’s intranet and in the coffee rooms of the factory workers. Also, employees who visited the occupational physician of the company for a routinely health check received information about the study. Participation was voluntary; employees who were interested in the study received an informed consent form and an online survey that could be accessed after providing consent. This survey was hosted by Castor, a highly secured, cloud-based electronic data capture platform [22]. A reminder was sent by email to employees who did not complete the survey.

Employees could participate in the study regardless of their position in the company. Eligible employees were 40–68 years, spoke Dutch fluently, and completed the informed consent form. The informed consent included permission to complete the survey, to request the OEC outcome at VeiligheidNL, and to use the outcomes for this study.

### Outcome measures

#### Need for recovery

NFR was the primary outcome measure of the study, which was assessed with the NFR-scale score of the Questionnaire on the Experience and Evaluation of Work version 2.0 (QEEW2.0) [23]. This scale consists of six statements concerning the short-time effects of a day of work, such as "I find it hard to relax at the end of a working day" and "When I get home, people should leave me alone for some time". All statements have four response options, respectively "always", "often", "sometimes", and "never". The sum-score of the scale can be converted to a scale score ranging from 0 to 100. A higher score indicates higher NFR.

#### Listening effort

LE was the secondary outcome of the study, which was assessed with the Amsterdam Checklist for Hearing and Work (ACHW). Employees were asked how much effort and concentration it takes to fulfill six hearing activities at their workplace, specifically detecting sounds, distinguishing sounds, communicating in quiet, communicating in noise, localizing sounds and being exposed to loud sounds. According to Van der Hoek-Snieders et al. (2020), we calculated a sum-score of the items. This score can range between 0 and 18.

### Determinants

#### Hearing status

The ability to understand speech in noisy situations was assessed with the OEC; an internet-based speech-in-noise hearing screening that is hosted by VeiligheidNL [24]. The stimuli consist of a closed set of eight equally intelligible CVC words that are presented in a stationary low-pass filtered masking noise. During this tests, the stimuli and the noise are presented via headphones. After presentation of the word, the corresponding button on a computer or telephone screen should be identified. The stimulus level decreases with 2 dB after every correct response and increases with 2 dB after every incorrect response. The first stimulus is presented at a signal-to-noise ratio (SNR) of 0 dB SNR and is followed by an up-down procedure with a 2 dB step size. After the first incorrect response, 20 stimuli are presented. The outcome of the OEC can be expressed as the speech reception threshold (SRT), which is calculated by averaging the SNR of the last ten stimuli. The SRT is determined for the right and the left ear separately. For clinical purposes, the screening outcome is pass if the SRT of at least one ear is − 14.9 or lower. For our statistical analyses, the SRT values of the better ears were used, because the better ear was expected to be the best predictor for hearing disability [25]. High sensitivity and moderate specificity have been established for the OEC, taking pure-tone audiometry as the reference standard [24].

#### Perceived workplace noise

The workplace noise intensity perceived by the employees was assessed by a visual analogue scale ranging from 0 (no noise at all) to 100 (very noisy).

### Confounders

Since NFR is a complex construct that is influenced by personal and work-related factors [9, 26], we controlled for several personal and work-related factors in the analysis. The personal factors include gender, age, educational level and perceived health status. Age was measured continuously. Educational level was categorized into three groups, respectively low (primary education, lower general secondary education, and preparatory secondary vocational education), medium (intermediate vocational training and general secondary education) and high (higher vocational education and university education). Perceived health status had four response categories, respectively very good, good, fair, and bad.

The work-related factors include the pace and amount of work, job variety, and work pleasure. These factors were measured with the three scales of the QEEW 2.0. These scales consist of statements measured on a five-point Likert scale. The sum-score of the scales can be converted to a scale score ranging from 0 to 100 with higher scores representing more unfavorable scores. A higher score indicates higher pace and amount of work, less job variety, and less work pleasure. The scale pace and amount of work consists of six statements, for example “Do you have to hurry”. The scale job variety consists of 4 statements, including “Do you have enough variety in your work”? The scale work pleasure consists of five statements, including “I enjoy my work”.

### Statistical analysis

Descriptive statistics was generated to report the characteristics of the study population. Distributions of all variables were examined and checked on normality. To gain insight into possible multicollinearity, we computed Pearson correlation coefficients between all dependent, continuous variables and the outcome measures and bi-serial correlation coefficients between all dependent, dichotomous/ordinal variables and the outcome measures. Correlations between the dependent variables were allowed if they were lower than 0.60.

Hierarchical multiple regression analyses were conducted to assess the relationship between understanding speech in noise and NFR (primary outcome measure) and LE (secondary outcome measure). For both outcomes, predictor variables were included in three blocks (forced entry). For each block, we calculated the change in amount of variance in the outcome variable that is explained by the dependent variables and the contribution of the individual predictors.

The blocks of independent variables were the same for the primary and secondary outcome measure. The first block included the possible confounders. A main effect of hearing status was added in the second block. In the third block, an interaction term was added, respectively the interaction between hearing status and the perceived noise level. A significance level of 0.05 was used.

## Results

In total, 180 employees were interested to participate in the study of which 8 did not respond on the study information and 29 could not be included because they were aged below 40. This resulted in a study population of 143 employees. Table 1 shows their demographics.

**Table 1:** Demographic characteristics of the study population (*N* = 143)

Variable	Category	*N*	%
Gender	Male	107	74.8
	Female	36	25.2
Age	40–50	50	35
	50–60	73	51
	> 60	20	14
Educational level	Primary school	4	2.8
	Lower vocational	10	7.0
	General intermediate	9	6.3
	Vocational education and training	48	33.6
	General secondary	8	5.6
	Higher vocational	45	31.5
	University	19	13.3
Perceived health status	Very good	25	17.5
	Good	103	72.0
	Fair	14	9.8
	Bad	1	0.7
Hearing protection	Yes	93	65.0
	No	50	35.0
Hearing aids	Yes	4	2.8
	No	138	97.2

The majority (75%) of the study population was male, mean age was 53, and their educational level varied from primary school to university. Most employees (89%) reported their health condition to be very good or good, and the others reported their health condition to be fair (10%) or bad (1%). Most employees (65%) used hearing protection at work. Only a few employees (4) were hearing aid users.

A normal distribution was confirmed for all continuous variables. The mean outcome of the hearing screening was -16.0 (range − 19.6; − 2.5). The outcome of the hearing screening was pass for 98 (68.5%) employees and fail for the other 45 employees (31.5%). Considering the best ears only, the mean outcome of the hearing screening was -17.0 (range − 19.6; − 10.0) and the outcome of the hearing screening was fail in 19 employees (13.3%).

A normal distribution was confirmed for all variables. Table 2 shows the relationships between the variables. The correlations between all independent variables were below 0.60 and thus there was no indication of multicollinearity. Hearing status was significantly associated with LE (*r* = 0.39, *p* < 0.01), but not with NFR (*r* = 0.04, *p* = 0.74).

**Table 2:** Means, standard deviations and Pearson correlations between personal factors, work-related factors, hearing status, listening effort, and need for recovery (*N* = 143)

Measure	M	SD	2	3	4	5	6	7	8	9	10	11
1. Gender	–	–	– 0.12	0.22**	0.06	0.22**	0.04	– 0.01	0.14	– 0.30**	0.14	– 0.10
2. Age	53.1	6.9		– 0.14	– 0.03	– 0.14	– 0.08	– 0.07	0.25	0.08	– 0.16	– 0.12
3. Educational level	–	–			– 0.20*	0.28**	0.30**	– 0.06	– 0.28*	– 0.43**	0.27**	0.03
4. Health status	–	–				– 0.05	– 0.25**	0.16	0.20	0.15	0.10	– 0.02
5. Pace/amount of work	36.4	15.1					0.18*	0.21*	0.01	– 0.27**	0.43**	0.04
6. Job variety	62.3	17.0						– 0.31**	– 0.24	– 0.05	0.12	0.05
7. Work pleasure	24.6	17.3							0.25	– 0.05	0.34**	0.03
8. Hearing status	-17.0	2.1								– 0.06	0.04	0.39**
9. Noise level	36.0	24.3									– 0.18*	0.12
10. NFR	22.7	15.3										0.33**
11. LE	2.5	2.2										

*p < 0.05; **p < 0.01

Gender, age, educational level, health status, pace/amount of work, job variety, and work pleasure were entered as predictors in Block 1 and as control variables in Block 2.

The results of the hierarchical multiple regression analysis are presented in Table 3. It shows that hearing status nor the interaction between hearing status and the perceived noise level was significantly associated with NFR. Hearing status was significantly associated with LE, but the interaction between hearing status and the perceived noise level was not.

**Table 3:** Results of the hierarchical multiple regression analysis of the factors associated with Need For Recovery (NFR) and subjective listening effort (LE)

Outcome	Block	Predictors	*p*
NFR	1	Gender	ns
		Age	ns
		Educational level	0.02
		Health status	0.04
		Pace/amount of work	< 0.01
		Job variety	ns
		Work pleasure	< 0.01
	2	Hearing status	ns
	3	Hearing status	ns
		Hearing status x perceived workplace noise	ns
LE	1	Gender	ns
		Age	ns
		Educational level	ns
		Health status	ns
		Pace/amount of work	ns
		Job variety	ns
		Work pleasure	ns
	2	Hearing status	0.02
	3	Hearing status	0.03
		Hearing status x perceived workplace noise	ns

Regarding the outcome NFR, the change in explained variance was significant for the first block (R square change = 0.314, *p* < 0.01), but not for the second block (R square change = 0.000, *p* = 0.96) nor the third block (R square change = 0.001, *p* = 0.69). The percentage explained variance for all three blocks was 27%.

For the outcome LE, the change in explained variance was significant when the second block was included (R square change = 0.07, *p* > 0.01), but not for the first block (R square change = 0.031, *p* = 0.74) nor the third block (R square change = 0.015 *p* = 0.14). The model including the possible confounders explained only 3% of the variance. The second model, after inclusion of hearing status, explained 10% of the variance. The third model, after inclusion of hearing status and the interaction between hearing status and the perceived noise level, explained 12% of the variance.

## Discussion

Many researchers have assessed the reliability and validity of speech-in-noise screening tests for identifying hearing loss, but it was unclear if poorer screening outcomes are also associated with higher LE and higher NFR. We found that hearing status was significantly associated with LE, but not with NFR in a population of employees of a manufacturing company. The associations did not depend on the perceived noise level at the workplace.

The finding that poorer hearing is associated with higher LE during the performance of auditory job tasks is in line with earlier studies in clinical populations [7, 9]. It suggests that the performance of auditory job tasks can be more demanding for employees with poorer hearing. It should however be noted that only weak-to-moderate associations were found in these studies: r = 0.39 in this study and *r* = 0.20, and *r* = 0.69 in the earlier studies, Ref. [7, 9], respectively. This implies that the effort it takes to perform auditory job tasks is only partly determined by hearing status. For example, cognitive load and speaker characteristics play a role according to the classification of Mattys et al. [27], such as pronunciation, disfluencies, and speech disorders. This might suggest that LE at work also depends on the complexity of the job task and on speaker characteristics of colleagues.

A non-significant association was found between hearing status and NFR. Earlier, mixed results were presented regarding the association between the ability to understand speech in noise and NFR [9, 10, 28]. A significant association was found in the cross-sectional study of Nachtegaal et al. [10] and in the longitudinal study of Van Leeuwen et al. [28]. These studies included adults with normal hearing and adults with various degree of hearing loss. No significant association was found in the cross-sectional analyses of Van der Hoek-Snieders et al. [9, 29]. They included employees that visited an audiological center, because of hearing complaints in their work situation. The degree of hearing loss was moderate in the majority of these employees. The mixed study results might be explained by the population differences between the studies, since an association is more likely to be demonstrated when there is larger variation in the degree of hearing loss.

Although it is assumed that the impact of poorer hearing on listening effort may be greater in noisy work environments [7, 10], we did not find a significant association between the perceived workplace noise and LE. Furthermore, no significant interaction effect was found between hearing status and perceived workplace noise in predicting hearing status. An explanation might be that—considering the OEC outcome—the vast majority of the study population is expected to be normal hearing. Possibly, small differences between normally-hearing employees are not associated with LE, even not in noisy work environments. Another explanation might be that hearing protection was used by 65% of the employees under study, since hearing protection is expected to reduce the hindrance of loud noises. The interaction between hearing status and perceived workplace noise for predicting LE and NFR should be assessed in a population with higher degree of hearing loss.

Some study limitations should be noted. There is a risk for selection bias, since employees voluntarily participated in this study. For example, feeling insecure about the hearing status might have been a reason to not participate in the study. Also, because there is currently no validated questionnaire available that measures LE during hearing related job activities, we used a non-validated questionnaire. Finally, we controlled for a broad spectrum of confounders. Nevertheless, we are not sure that we controlled for all relevant confounders since NFR is a complex construct. For example, the cognitive load and physical demands of employees’ job might have been relevant.

Considering the moderate association between hearing status and LE, the OEC is expected to inadequately predict subjective listening difficulties at the workplace at individual level. The predictive value of the OEC for high NFR is expected to be even poorer. Although the OEC is an appropriate instrument to assess employees’ ability to understand speech in noisy environments [18, 21], the added value of occupational hearing screening for the identification of subjective listening difficulties and/or difficulties with work participation is modest. Occupational hearing screening might be valuable to rule out hearing loss as an underlying cause of listening difficulties or difficulties with work participation, for example in employees that present with complaints of fatigue after work. This should be investigated by future research.

## Conclusion

Our results confirm that poorer hearing is associated with higher LE, but not with higher NFR. These associations were unrelated to the perceived noise level at the workplace. Therefore, the value of occupational hearing screening appears to be primarily in early identification of hearing loss in employees, but not in the identification of subjective listening difficulties and/or difficulties with work participation. Future research should investigate the value of occupational hearing screening for identifying hearing loss as a hidden cause of work participation difficulties.

## Data Availability

The data that support the findings of this study are available from the corresponding author, HH, upon reasonable request.
